# PRMT7 deficiency causes dysregulation of the HCN channels in the CA1 pyramidal cells and impairment of social behaviors

**DOI:** 10.1038/s12276-020-0417-x

**Published:** 2020-04-08

**Authors:** Seul-Yi Lee, Tuan Anh Vuong, Hyun-Kyung So, Hyun-Ji Kim, Yoo Bin Kim, Jong-Sun Kang, Ilmin Kwon, Hana Cho

**Affiliations:** 1 Department of Physiology, Sungkyunkwan University School of Medicine, Suwon, Korea; 2 Single Cell Network Research Center, Sungkyunkwan University School of Medicine, Suwon, Korea; 3 Department of Molecular Cell Biology, Sungkyunkwan University School of Medicine, Suwon, Korea; 4 Department of Anatomy and Cell Biology, Sungkyunkwan University School of Medicine, Suwon, Korea

**Keywords:** Excitability, Neurophysiology

## Abstract

HCN channels regulate excitability and rhythmicity in the hippocampal CA1 pyramidal cells. Perturbation in the HCN channel current (*I*_h_) is associated with neuropsychiatric disorders, such as autism spectrum disorders. Recently, protein arginine methyltransferase 7 (PRMT7) was shown to be highly expressed in the hippocampus, including the CA1 region. However, the physiological function of PRMT7 in the CA1 neurons and the relationship to psychiatric disorders are unclear. Here we showed that PRMT7 knockout (KO) mice exhibit hyperactivity and deficits in social interaction. The firing frequency of the CA1 neurons in the PRMT7 KO mice was significantly higher than that in the wild-type (WT) mice. Compared with the WT CA1 neurons, the PRMT7 KO CA1 neurons showed a more hyperpolarized resting potential and a higher input resistance, which were occluded by the *I*_h_-current inhibitor ZD7288; these findings were consistent with the decreased *I*_h_ and suggested the contribution of *I*_h_-channel dysfunction to the PRMT7 KO phenotypes. The HCN1 protein level was decreased in the CA1 region of the PRMT7 KO mice in conjunction with a decrease in the expression of *Shank3*, which encodes a core scaffolding protein for HCN channel proteins. A brief application of the PRMT7 inhibitor DS437 did not reproduce the phenotype of the PRMT7 KO neurons, further indicating that PRMT7 regulates *I*_h_ by controlling the channel number rather than the open probability. Moreover, shRNA-mediated PRMT7 suppression reduced both the mRNA and protein levels of SHANK3, implying that PRMT7 deficiency might be responsible for the decrease in the HCN protein levels by altering *Shank3* expression. These findings reveal a key role for PRMT7 in the regulation of HCN channel density in the CA1 pyramidal cells that may be amenable to pharmacological intervention for neuropsychiatric disorders.

## Neuroscience: Finding the brakes in the autistic brain

Disrupted expression of an ion channel that helps stabilize brain cell activity contributes to behavioral symptoms in mice resembling those seen in autism spectrum disorders (ASDs). Nerve cell firing depends on the right balance of ions inside and outside cells, and a channel protein called HCN helps establish ionic conditions that prevent excessive activity. Researchers led by Hana Cho and Ilmin Kwon of the Sungkyunkwan University School of Medicine, Suwon, South Korea have demonstrated that mice lacking another protein called PRMT7 exhibit reduced numbers of HCN channels in brain structures known to be affected in animal models of ASDs. These mice exhibit hyperactivity and social anxiety, presumably as a consequence of poor regulation of nerve cell firing. The authors propose that this PRMT7-HCN pathway may offer a fruitful target for the development of neuropsychiatric therapies.

## Introduction

Neuronal signaling within a region and at a circuit level is affected by intrinsic excitability and the distribution of ion channels. Dysfunction of ion channels can lead to abnormal excitability and subsequent neuronal dysfunction. HCN channels are present throughout the brain and modulate neuronal excitability and activity^1^ via the hyperpolarization-activated current, *I*_h_ (also known as h current), consisting of sodium and potassium cations^2^. Four subunits (HCN 1–4) of the HCN channels that conduct the *I*_h_ currents have been identified^2–5^. HCN channels are activated by hyperpolarized states >−40 mV, increasing their activation as cells become more hyperpolarized, and do not display inactivation^6^. Since HCN channels are open at hyperpolarized states >−40 mV, *I*_h_ is partially activated under physiological, “resting” conditions, providing constant depolarization of the membrane potential^7–9^. Thus *I*_h_ is thought to function as a stabilizing negative-feedback loop that responds to alterations in membrane potential^6,10^. In addition to its depolarizing role, *I*_h_ exerts a shunting effect on excitable cells: being open at subthreshold potentials, *I*_h_ reduces the input resistance of the membrane, thus dampening the ability of incoming inputs to alter membrane voltage. These factors allow HCN channels to influence the rhythmicity of neuronal networks and to control the integration of cell signaling and firing activity.

HCN channels are highly expressed in pyramidal neurons in the hippocampal CA1 region, where they serve to modulate network excitability to integrate incoming signals, normalize temporal summation, and mediate the propagation of information by dampening Ca^2+^ signaling^11–13^^.^ The HCN channel in CA1 not only has essential roles in the modulation of neuronal excitability under physiological conditions but also plays an important but complex role in neuropsychiatric diseases. Kim and colleagues^14^ showed that HCN1 in CA1 was increased with chronic mild stress and that short hairpin RNA (shRNA) knockdown of HCN1 in CA1 led to a reduction in anhedonia-like behavior and an increase in antidepressant-like behavior. In addition, hyperexcitability of the hippocampal CA1 pyramidal neurons was observed in a rat model of autism spectrum disorders (ASDs)^15^. Furthermore, autism-associated SHANK3 haploinsufficiency causes *I*_h_ channelopathy in hippocampal neurons^16^.

Protein methylation, along with phosphorylation, controls various cellular functions through regulation of signaling pathways or gene expression^17–20^. Protein arginine methyltransferases (PRMTs) catalyze the transfer of a methyl group to the arginine residues of target proteins^21,22^. Among the nine characterized members, PRMTs can be classified into three types based on their catalytic activity. Type I PRMTs (PRMT1, PRMT2, PRMT3, PRMT4, PRMT6, and PRMT8) catalyze the formation of asymmetrically dimethylated arginine residues on substrate proteins, and type II PRMTs (PRMT5 and PRMT9) catalyze symmetrically dimethylated arginine residues. Finally, the type III PRMTs, including PRMT7, catalyze monomethylated and symmetrically dimethylated arginine residues. Recently, we and others have demonstrated the physiological roles of PRMT7 in the control of neural differentiation and neuronal ion channels^23,24^. Dhar et al. showed that PRMT7 blocks the MLL4-mediated differentiation in the human progenitor cell line NT2/D1^23^. We showed that PRMT7 is highly expressed in the hippocampal CA1 region and the dentate gyrus (DG) and that PRMT7 regulates the excitability of DG granule cells via the Na^+^ leak channel NALCN. However, the physiological function of the PRMT7 protein in CA1 and its relationship to psychiatric disorders have not been investigated.

In this study, we showed that mice null for PRMT7 (PRMT7 knockout (KO)) exhibit hyperactivity and impairment of social behaviors. Patch clamp recordings confirmed that the intrinsic excitability of the PRMT7 KO CA1 pyramidal cells was significantly increased compared to that of the wild-type (WT) CA1 pyramidal cells. Compared with the WT CA1 neurons, the PRMT7 KO CA1 neurons showed a more hyperpolarized resting potential and a higher input resistance, which were occluded by the *I*_h_-current inhibitor ZD7288; these findings were consistent with the decreased *I*_h_ and suggested the contribution of *I*_h_-channel dysfunction to the PRMT7 KO phenotypes. The protein level of the HCN channel subunit HCN1 was lower in the PRMT7 KO CA1 region than in the control WT region, with a concomitant decrease in the gene expression of SHANK3, a scaffolding protein for the HCN channel. These results indicated that PRMT7 modulates *I*_h_ via controlling the HCN1 protein level. Moreover, a brief application of the PRMT7 inhibitor DS437 did not affect the HCN1 and HCN2 channel activities in the HEK-293T cells as well as the neuronal excitability of the CA1 pyramidal cells, further indicating that PRMT7 deficiency reduces *I*_h_ by a reduction in channel number rather than the open probability. Taken together, our data suggest that the induction of social deficits is associated with hyperexcitability in CA1 pyramidal neurons, which could be attributable to changes in *I*_h_-channel function. This finding provides a novel mechanism for the modulation of the HCN channel, suggesting a potential therapeutic target, and suggests a critical window of intervention for neuropsychiatric diseases.

## Materials and methods

### Animal studies

Prmt7 mice were originally purchased from the Sanger Institute. Mice were backcrossed onto C67BL/6J background for at least six generations and maintained on C67BL/6J background, and wild-type littermates were used as controls for the phenotype studies of the PRMT7 knockout mice^25^. All animal experiments were approved by the Institutional Animal Care and Use Committee (IACUC) of Sungkyunkwan University School of Medicine (IACUC2019-07-11-3).

The social approach test was performed as described previously^26^. A three-chambered plastic box was used to test sociability for social novelty. First, for habituation, the mice were first placed in the middle chamber and allowed to explore for 10 min. During habituation, each of the two side chambers contained an empty cylindrical cage. Following a 10-min habituation, for the sociability test, an unfamiliar mouse (social object, age-matched same-sex mouse) enclosed in a cylindrical cage was placed in one of the side chambers; an empty cylindrical cage was placed in the other side chamber, and then the subject mice were allowed to explore for 10 min. In sociability tests, the time spent exploring each object in the side chambers was analyzed.

The open-field box was made of white plastic (40 cm × 40 cm × 40 cm), and the open field was divided into a central field (center, 20 cm × 20 cm) and an outer field (periphery). Individual mice were placed in the periphery of the field, and the paths of the animals were recorded with a video camera. The total distance traveled for 30 min and the time spent in the central area for the first 5 min period were analyzed using a program (EthoVision, Noldus, USA).

The maze was made of plastic and consisted of two white open arms (25 cm × 8 cm), two black enclosed arms (25 cm × 8 cm × 20 cm), and a central platform (8 cm × 8 cm × 8 cm) in the form of a cross. The maze was placed 50 cm above the floor. The mice were individually placed in the center with their heads directed toward one of the closed arms. The total time spent in each arm or in the center and the total number of entries into each arm were analyzed by video monitoring for 5 min. Only when all four paws crossed from the center into an arm, it was counted as an arm entry and used for measuring the amount of time spent in each arm.

The light and dark test box had dimensions of 12 cm × 30 cm × 20 cm for the light chamber (∼600 lux) and 14 cm × 13 cm × 20 cm for the dark chamber (∼5 lux). The mice were individually placed in the center of the light chamber and allowed to freely explore the whole apparatus for 10 min. The time spent in the light chamber was analyzed.

### Brain slice preparation and recording

Brain slices were prepared from the male KO mice and the WT littermates at 5–8 weeks of age. The mice were decapitated after being anesthetized with pentobarbital sodium, and the whole brain was quickly removed from the skull and chilled in artificial cerebrospinal fluid (aCSF) at 4 °C. Transverse hippocampal slices (350-μm thick) were prepared with a vibratome (VT1200S, Leica, Nussloch, Germany). The slices were incubated at 34 °C for 30 min and were thereafter maintained at 32 °C until in situ slice patch recordings and fluorescence microscopy were performed. Hippocampal pyramidal cells from the CA1 were visualized using an upright microscope equipped with differential interference contrast optics (BX51WI, Olympus, Tokyo, Japan). Whole-cell current clamp techniques were used to measure the excitability of the CA1 pyramidal cells. The pipette solution contained (in mM): 143 K-gluconate, 7 KCl, 15 HEPES, 4 MgATP, 0.3 NaGTP, 4 Na-ascorbate, and 0.1 EGTA with the pH adjusted to 7.3 with KOH. The bath solution (or aCSF) for the control experiments contained the following (in mM): 125 NaCl, 25 NaHCO_3_, 2.5 KCl, 1.25 NaH_2_PO_4_, 2 CaCl_2_, 1 MgCl_2_, 20 glucose, 1.2 pyruvate, and 0.4 Na-ascorbate, with a pH 7.4 when saturated with carbogen (95% O_2_ and 5% CO_2_). All bath solutions included 50 μM AP5, 10 μM CNQX, and 20 μM bicuculline to block all excitatory (AMPA and NMDA receptors) and inhibitory (GABA_A_ receptors) synaptic inputs. The perfusion rate of the bathing solution and the volume of the recording chamber for the slices were 2.2 ml/min and 1.2 ml, respectively. Patch pipettes with a tip resistance of 3–4 MΩ were used. The series resistance (*R*_s_) after establishing the whole-cell configuration was between 10 and 15 MΩ. The electrophysiological recordings were made in somata with a MultiClamp 700B amplifier paired with Digidata 1550 A digitizer (Molecular Devices, CA, USA), at a sampling rate of 20 kHz and low-pass filtered at 1 kHz (Bessel). The experiments were performed at 32 ± 1 °C. The following parameters were measured: (1) the resting membrane potential (RMP), (2) the action potential (AP) threshold current (current threshold for single AP generation, 100 ms duration), (3) the input resistance (*R*_in_, membrane potential changes (*V*) for a given hyperpolarizing current (35 pA, 600 ms) input), (4) the AP threshold potential, and (5) the *F*–*I* curve (firing frequencies (*F*) against the amplitude of the injected currents (*I*); 0–250 pA). We excluded data for analysis when the series resistance exceeded 20 MΩ or when the RMP was more positive than −60 mV.

### Cell culture, transfection, and recording

HEK-293T cells (ATCC, VA, USA) were maintained in Dulbecco’s modified Eagle’s medium (Gibco-BRL, CA, USA) supplemented with 10% fetal bovine serum at 37 °C in 5% CO_2_. For transient transfections, the cells were transfected using Effectene (Qiagen, Hilden, Germany), and green fluorescent protein was used as a reporter to label the transfectants. The HCN currents from the HEK-293T cells were measured with the whole-cell patch clamp technique. Voltage clamp was performed using an EPC-10 amplifier (HEKA Instruments, Lambrecht/Pfalz, Germany) at a sampling rate of 10 kHz filtered at 1 kHz. Data were acquired using an IBM-compatible computer running Patchmaster software (HEKA Instruments, Lambrecht/Pfalz, Germany). The patch pipettes were pulled from borosilicate capillaries (HilgenbergGmbH, Malsfeld, Germany) using a Narishige puller (PC-10, Narishige, Tokyo, Japan). The patch pipettes had a resistance of 2–3 MΩ when filled with the pipette solution containing (in mM) 135 K-aspartate, 2 MgCl_2_, 10 HEPES, 0.1 Na-GTP, 4 Mg-ATP, 1 CaCl_2_, and 3 EGTA, with a pH of 7.2 adjusted with KOH. The normal external solution was as follows (in mM): 143 NaCl, 5.4 KCl, 5 HEPES, 0.5 NaH_2_PO_4_, 11.1 glucose, 0.5 MgCl_2_, and 1.8 CaCl_2_, with a pH of 7.4 adjusted with NaOH. The pipette capacitance was compensated after the formation of a gigaohm seal. Access resistance was typically 2.8–3.2 MΩ. The perfusion system was a homemade 100-ml perfusion chamber through which solution flowed continuously at 5 ml/min. The currents from HEK-293T cells were studied by holding the cell at −40 mV, and 2-s steps from −140 to +20 mV in 10-mV increments were applied, followed by 1-s pulses to −40 mV. All recordings were carried out at room temperature. Currents were measured in a steady state at the end of the test pulses and were normalized with respect to the cell capacitance.

The currents were analyzed and fitted using the Patchmaster (HEKA Instruments, Lambrecht/Pfalz, Germany) and Origin (ver. 6.0, Microcal, MA, USA) softwares. All values are given as mean ± standard error. The *I*/*V* relationship was obtained by plotting the outward current at the end of a 1-s test pulse as a function of the test potential.

### Primary neuronal cultures from mice

Primary hippocampal neurons were isolated from the P0 mice as described previously^27^. Briefly, the hippocampal samples from the P0 mice were dissociated by trypsin digestion and plated on poly-L-lysine- and laminin-coated glass coverslips using neurobasal plating media (Neurobasal media containing B27 supplement; 1× GlutaMAX, 1× penicillin/streptomycin, 1 mM HEPES, 10% heat-inactivated donor horse serum). The next day, half of the cell culture medium was removed and replaced with the same volume of Neurobasal Feeding Medium (Neurobasal medium containing B27 supplement, 1× GlutaMAX, 1× penicillin/streptomycin, 1 mM HEPES). The medium replacement was conducted every 2 days until use.

### Immunofluorescence staining, immunoblotting, and RNA analysis

Immunostaining was performed as previously described^28^. Briefly, 8-week-old mice were fixed with 4% paraformaldehyde (PFA), and the dissected brains were further fixed with 4% PFA overnight at 4 °C. Then the brain was dehydrated through a sucrose series followed by cryoembedding and sectioning at 10-μm thickness on a cryostat microtome (Leica). Immunostaining was carried out as previously described^29^. Briefly, the brain sections were processed through antigen retrieval with 10 mM sodium citrate (pH 6.0), blocked, and incubated with primary antibodies overnight at 4 °C. Confocal microscopy was performed at Sungkyunkwan University School of Medicine Microscopy Shared Resource Facility with a Zeiss LSM-710 Meta confocal microscope.

Immunoblot analysis was performed as previously described^30,31^. Briefly, the brain tissues were lysed in RIPA buffer (iNtRON Biotechnology, Korea) containing complete protease inhibitor cocktail (Roche), followed by sodium dodecyl sulfate-polyacrylamide gel electrophoresis. Immunoprecipitation was performed as described elsewhere^32^. Briefly, the HEK-293T cells were transfected with the indicated plasmids using Lipofectamine 2000 reagents. The transfected cells were lysed in lysis buffer with 1% Triton X-100. Then the cell lysates were immunoprecipitated with 1 μg of primary antibodies or control IgG at 4 °C overnight followed by incubation with protein A/G agarose beads (Roche). The precipitates were washed and analyzed by immunoblotting. The primary antibodies used in the present study were HCN1 (GeneTex, CA, USA), HCN2 (Novus Biotechnology, CO, USA), PRMT5 (Cell Signaling Technology, MA, USA), PRMT7, HSP90, SHANK3 (Santa Cruz Biotechnology, TX, USA), β-tubulin (Sigma, MO, USA), HA (Abcam, Cambridge, UK), and SYM10 (Cell Signaling Technology, MA, USA).

Quantitative reverse transcriptase-PCR analysis was carried out as previously described^28^. Briefly, the tissues were homogenized by FastPrepR-24 (MP Biomedicals, CA, USA) and extracted with an Easy-Spin Total RNA Extract Kit (iNtRON, MA, USA). The data were normalized to actin. The primer sequences used in this study are as follows: *Hcn1* (ACATGCTGTGCATTGGTTATGGCG and AACAAACATTGCGTAGCAGGTGGC), *Hcn2* (ACTTCCGCACCGGCATTGTTATTG and TCGATTCCCTTCTCCACTATGAGG), *Shank3* (GTTGCGAGCTGCTTCTCCAT and GCGCAACTCTCCTGGTTGTA) and *Actin* (GTCCCTGACCCTCCCAAAAG and GCTGCCTCAACACCTCAACCC).

### Statistical analysis

All data analysis and curve fittings were performed using Origin 6.0 and Igor Pro. Values are reported as the mean ± S.D. or the mean ± S.E.M., as indicated. Student’s *t* test was used for statistical significance, and *p* values are given in the figure legends.

## Results

### The PRMT7 KO mice show impaired social behavior

**Fig. 1:**
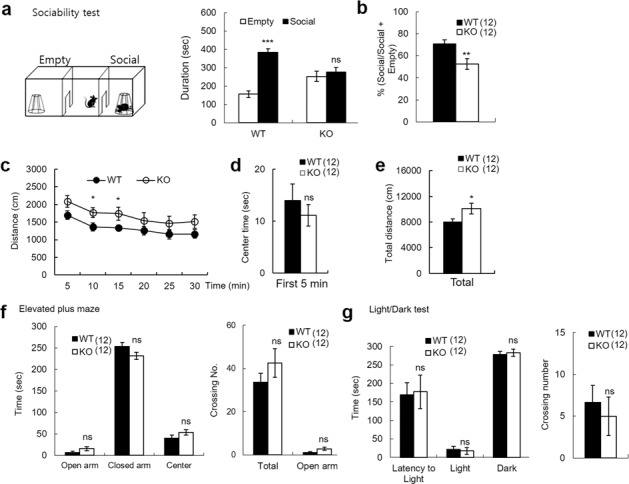
Decreased social interaction but normal locomotion and anxiety-like behavior in the adult PRMT7 KO mice. **a**, **b** Social approach (three-chamber) test. **a** illustrates the time spent interacting with an empty cup or a cup with a strange mouse inside, which is shown for each genotype. Paired Student’s *t* test comparing “mouse” to “empty” as a measure of sociability within each group. ***p < 0.001; ns, not significantly different. **b** illustrates the preference for the strange mouse, calculated as [(time spent exploring stranger mouse)/(total time spent exploring stranger mouse and empty cup)] × 100%. **p < 0.01; Student’s *t* test. **c**–**e** Normal locomotor activity of the PRMT7 KO mice (3 months) in open-field tests, as shown by the distance moved (**c**), the time spent in the center region of the open-field arena (**d**), and the total distance moved for 30 min (**e**). *n* = 12 for WT and 12 for KO. *p < 0.05; ns, not significantly different; Student’s *t* test. **f** Normal anxiety-like behavior o**f** the PRMT7 KO mice (3 months) in the elevated plus-maze test. *n* = 12 for WT and 12 for KO. ns, not significantly different, Student’s *t* test. **g** Normal anxiety-like behavior of the PRMT7 KO mice (3 months) in the light/dark test. *n* = 12 for WT and 12 for KO. ns not significantly different; Student’s *t* test.

To determine the impacts of PRMT7 deletion on behaviors, we subjected the PRMT7 KO mice to a battery of behavioral tests. Because PRMT7 is highly expressed in the hippocampal CA1 region and has been shown to be associated with ASDs and depression^15,33^, we first tested whether the PRMT7 KO mice displayed social deficits. To assess the social interaction of the PRMT7 KO mice, we utilized the three-chamber social approach test, which has been extensively used in studies of sociality with various mouse lines^34^. The sociability phase of the test measures the preference of the subject for exploring either a novel adult male conspecific enclosed in a ventilated container (stranger) or an identical but otherwise empty container. This task is related to the tendency of autistic children to spend more time playing with an inanimate toy than be engaged in social interactions with other children^35^. In contrast to the WT mice, the PRMT7 KO mice spent less time exploring the novel mouse and did not show a preference for exploring the stranger versus the empty container (Fig. 1a, b). The reduced social interaction of the PRMT7 KO mice was not due to deficient ambulation within the arena, as the PRMT7 KO mice showed a slight increase in locomotor activity in an open-field test compared to the WT mice (Fig. 1c–e). The PRMT7 KO mice (*n* = 12) spent a similar amount of time in the center of the open-field box compared with the WT mice (*n* = 12) (Fig. 1d). However, the PRMT7 KO mice moved a longer distance than the WT mice (Fig. 1c). The total travel distance was significantly increased in the PRMT7 KO mice compared to the WT mice (*p* < 0.05; Fig. 1e). The PRMT7 KO mice showed normal anxiety-like behaviors in the elevated plus-maze (Fig. 1f) and the light/dark test (Fig. 1g). In addition, long-term video electroencephalographic (EEG) recording and monitoring of behavior and spontaneous EEG activity revealed that the KO mice did not exhibit any seizure activity (Supplementary Fig. 1). Collectively, these results suggest that PRMT7 deficiency specifically impairs social interaction in mice without altering the general anxiety levels or spontaneous seizures.

### Enhanced excitability and input resistance of the CA1 pyramidal cells in the PRMT7 KO mice

**Fig. 2:**
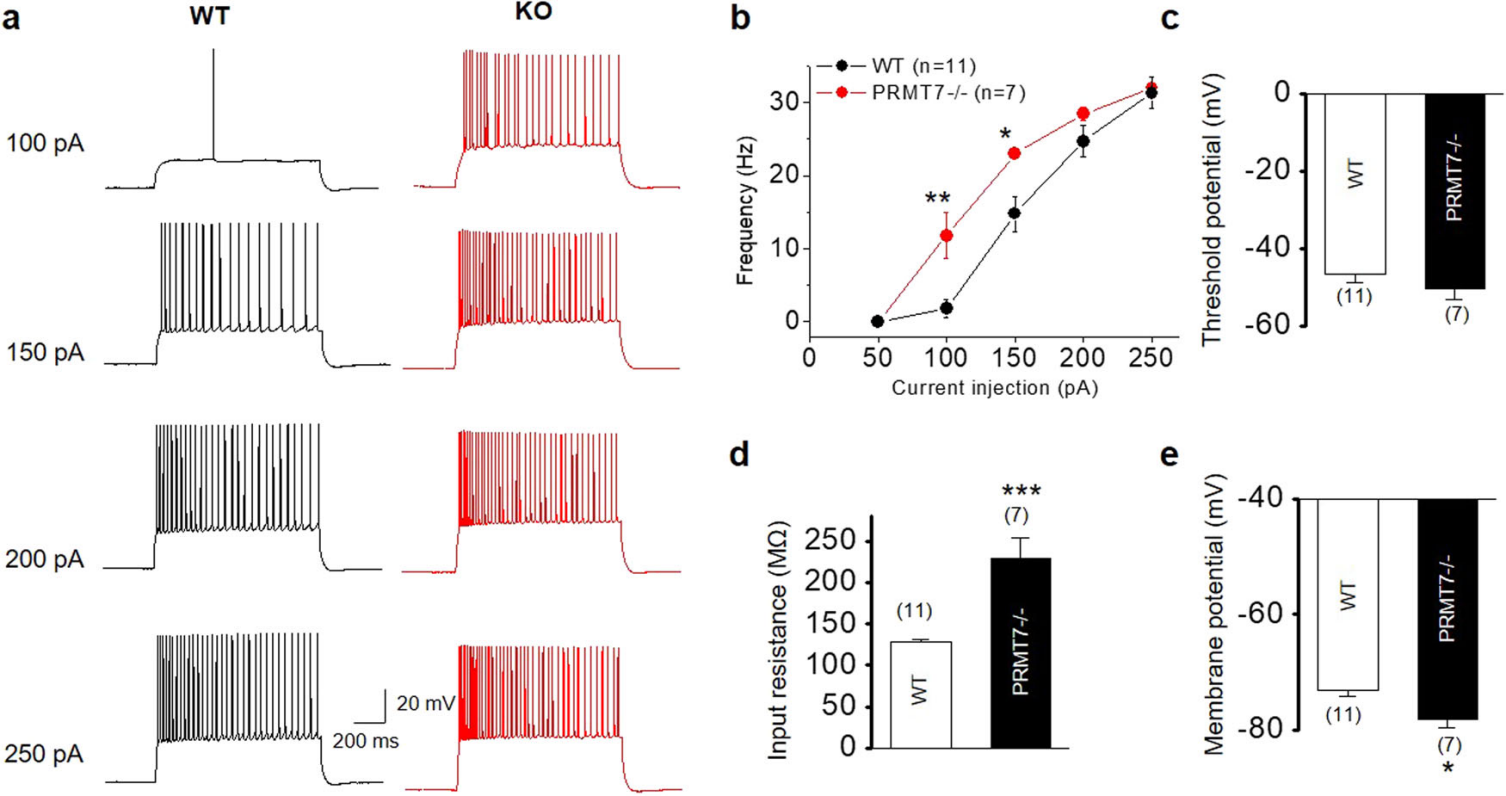
Increased firing frequency in the CA1 pyramidal cells from the PRMT7 KO mice. **a** Representative traces in the whole-cell current clamp recording in the mature WT and PRMT7 KO CA1 pyramidal cells in response to 1-s depolarizing current injection (from 100 to 250 pA, +50-pA increment, during 1-s). **b** The mean number of action potentials (AP no.) plotted against the eliciting currents. At all amplitudes, the mean ± S.E.M. AP no. is significantly higher in the PRMT7 KO pyramidal cells (red circles; *n* = 7) than in the WT pyramidal cells (black circles; *n* = 11). **c**–**e** The mean value of the threshold potential (**c**), the input resistance (**d**), and the resting membrane potential (**e**) in the WT and PRMT7 KO CA1 pyramidal cells. *p < 0.05; **p < 0.01; ***p < 0.001; Student’s *t* test.

The hippocampal CA1 region stores social memory, and dysfunction of the CA1 pyramidal neurons has been associated with impairments in social interactions and ASDs^15,33^. To identify the neural mechanisms underlying the impaired social interaction in the PRMT7 KO mice, we performed electrophysiological recordings of the CA1 pyramidal cells from the hippocampal slices obtained from the WT and KO mice. The CA1 pyramidal cells from the WT mice typically displayed tonic firing patterns in response to a 1-s square current pulse injection: AP frequency increased as the magnitude of the square pulse increased (Fig. 2a, left). The CA1 pyramidal cells of the PRMT7 KO mice showed a significantly higher AP frequency than those of the WT mice (Fig. 2b, right). The average AP frequency in response to a 100-pA depolarizing current in the WT pyramidal cells was 1.8 ± 1.3 Hz (*n* = 11), while it increased significantly to 13.7 ± 3.1 Hz (*n* = 7, *p* < 0.01) in the PRMT7 KO pyramidal cells (Fig. 2b). These data indicate that the deletion of the PRMT7 gene elevated the excitability of the hippocampal CA1 neurons. Since the threshold potential was not changed in the PRMT7 KO pyramidal cells (Fig. 2c), we measured the RMP and the input resistance of the CA1 neurons from the WT and PRMT7 KO mice. The analysis of the pooled results revealed that the PRMT7 deletion caused a large increase (~80%) in the input resistance (Fig. 2d). Moreover, the PRMT7 KO CA1 pyramidal cells exhibited a significant hyperpolarization in the RMP compared to the WT CA1 pyramidal cells (*p* < 0.05; Fig. 2e). Thus the hyperexcitability in the PRMT7 KO pyramidal cells might be attributable to an increased input resistance; the action on input resistance in this case compensates for the more hyperpolarized membrane voltage, an effect that should by itself decrease excitability.

### PRMT7 KO renders CA1 neurons hyperexcitable by impairing *I*_h_ currents

**Fig. 3:**
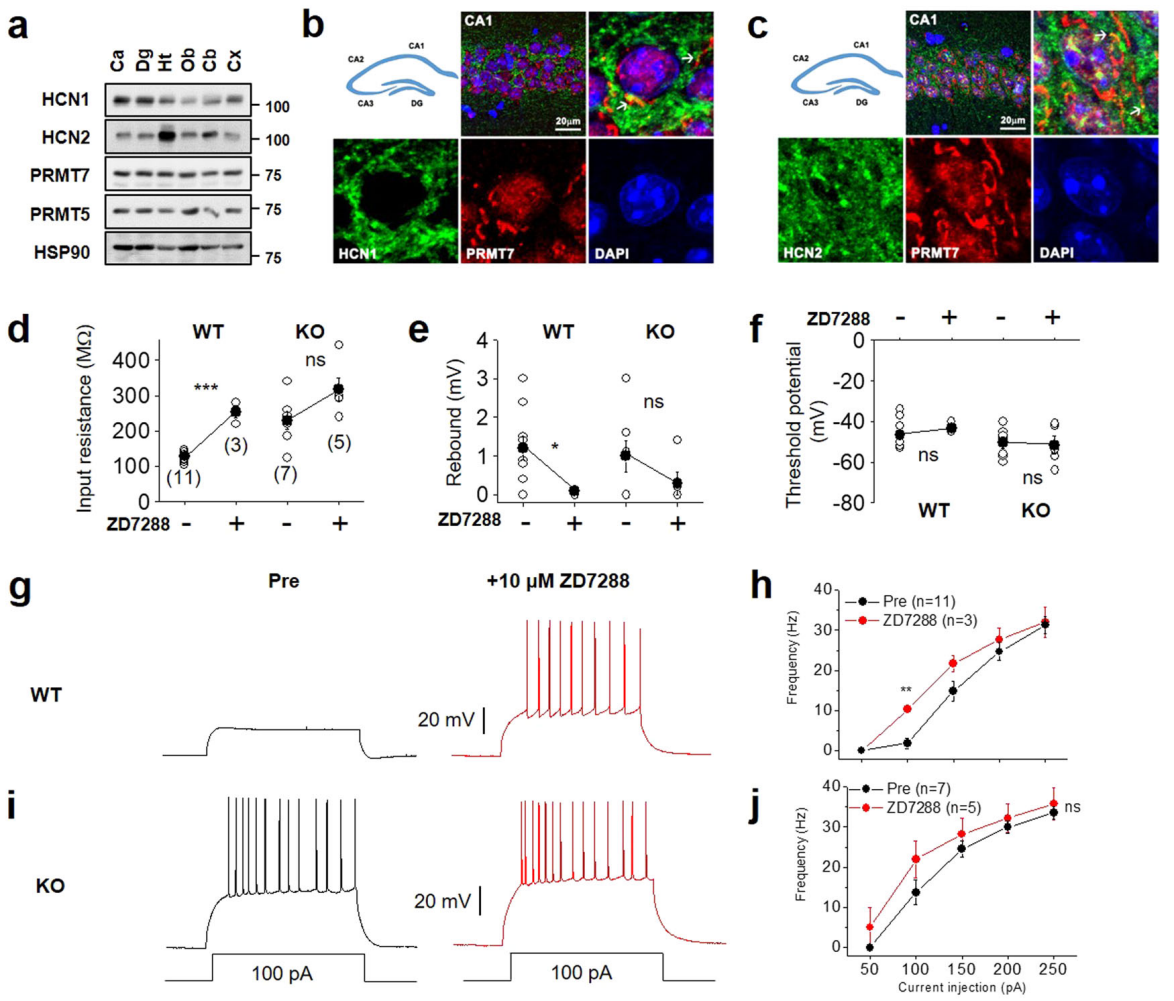
PRMT7 KO renders CA1 neurons hyperexcitable by impairing *I*_h_ currents. **a** Immunoblotting for the HCN1, HCN2, PRMT5, and PRMT7 proteins in various brain regions in 8-week-old mouse brains. Ca cornu ammonis, Dg dentate gyrus, Ht hypothalamus, Ob olfactory bulb, Cb cerebellum, Cx cerebral cortex. **b**, **c** Immunostaining of HCN1 (green, **b**), HCN2 (green, **c**), and PRMT7 (red) in the CA region of the 8-week-old mouse brains. Scale bar = 20 µm. **d**–**f** Changes in the input resistance (**d**), the rebound potential (**e**), and the threshold potential (**f**) in the WT and PRMT7 KO CA1 pyramidal cells in response to 10 μM ZD7288. Each dot represents an individual pyramidal cell. Closed circles represent the mean values (error bars; S.E.M). **g**–**j** The *I*_h_-current inhibitor ZD7288 increased firing frequency in the WT but not in the PRMT7 KO CA1 pyramidal neurons. **g**, **i** illustrate representative traces before and after 10 μM ZD7288 in the WT and KO pyramidal cells, respectively. Summarized data comparing the number of APs before and after the application of 10 μM ZD7288 in the WT (**h**) and PRMT7 KO pyramidal cells (**j**). *p < 0.05; **p < 0.01; ***p < 0.001; ns not significantly different; Student’s *t* test.

The input resistance of neurons depends at least in part on their ionic conductance and ion channels. We have previously reported that PRMT7 regulates NALCN in the DG region^24^. However, PRMT7 KO increased the DG excitability through NALCN activation and consequent membrane depolarization, which is inconsistent with an increase in the input resistance and the membrane hyperpolarization in the PRMT7-deficient CA1 neurons. Thus we concluded that NALCN does not have a role in PRMT7 KO CA1 hyperexcitability. Another candidate for the impaired membrane conductance as a cause of increases in the input resistance and the hyperpolarization of the RMP in the PRMT7 KO pyramidal cells is *I*_h_ currents. *I*_h_ currents depolarize membranes toward the AP threshold and reduce membrane resistance. All four HCN isoforms are expressed in the mammalian brain^1,36,37^. Of these four, HCN1 and HCN2 are the major isoforms in the hippocampal CA1 neurons^6^. We confirmed that the HCN1 and HCN2 subunits of the HCN channels are expressed in various brain areas, including the DG and the cornu ammonis (CA) of the hippocampus, similar to PRMT7 (Fig. 3a). The immunostaining of CA1 further showed that the PRMT7 and HCN proteins are coexpressed and partially colocalized in the CA1 pyramidal cells (Fig. 3b, c, marked with white arrows).

To assess the involvement of *I*_h_ in the increased excitability of the PRMT7 KO CA1 pyramidal cells, we compared the effects of the *I*_h_-current inhibitor ZD7288 on the CA1 neurons from the control WT mice and the PRMT7 KO mice (Fig. 3d–j). ZD7288 was applied for 4–5 min before switching back to the normal aCSF^38^. A larger change in input resistance is indicative of an increase in functional HCN channels. Similarly, the rebound slope is an indirect measure of HCN channels, where a more negative slope signifies more *I*_h_. At −70 mV, a membrane potential where HCN channels would normally be active, ZD7288 caused the input resistance to increase in the CA1 neurons from the WT mice (aCSF: 127.6 ± 4.2 MΩ (*n* = 11), ZD7288: 253.3 ± 17.6 MΩ (*n* = 3), unpaired *t* test, *p* < 0.001; Fig. 3d). The input resistance of the CA1 neurons from the PRMT7 KO mice was unaffected by ZD7288 (aCSF: 228.9 ± 25.1 MΩ (*n* = 7), ZD7288: 317.6 ± 33.5 MΩ (*n* = 5), unpaired *t* test, *p* > 0.05; Fig. 3d). Similarly, ZD7288 application decreased the rebound potential in the WT neurons (aCSF: 1.2 ± 0.3 mV (*n* = 11), ZD7288: 0.1 ± 0.04 mV (*n* = 3), unpaired *t* test, *p* < 0.05; Fig. 3e) but had a less dramatic effect on the KO neurons (aCSF: 1.0 ± 0.4 mV (*n* = 7), ZD7288: 0.3 ± 0.3 mV (*n* = 5), unpaired *t* test, *p* > 0.05; Fig. 3e). This decreased sensitivity of the rebound potential to ZD7288, similar to the smaller change in input resistance, is consistent with lower *I*_h_. ZD7288 had little effect on the threshold potentials of both the WT and PRMT7 KO CA1 neurons (Fig. 3f). We further examined the effect of ZD7288 on the firing frequency. The firing frequency in the WT CA1 neurons increased significantly after applying 10 μM ZD7288 (Fig. 3g, h). For example, the AP frequency in response to a 100-pA depolarizing current was 1.8 ± 1.3 Hz (*n* = 11) in the control and increased to 10.3 ± 0.3 Hz (*n* = 3) following the application of 10 μM ZD7288 (*p* < 0.01; Fig. 3h). This ZD7288-induced spiking was largely absent in the PRMT7 KO neurons (Fig. 3i, j). Before and after ZD7288 treatment, the AP frequency in response to a 100-pA depolarizing current was 13.7 ± 3.1 Hz (*n* = 7) and 22.0 ± 4.6 Hz (*n* = 5), respectively (*p* > 0.05; Fig. 3j). Thus these results showed that the PRMT7 KO CA1 pyramidal cells displayed a high firing rate at baseline, and their firing did not further increase during ZD7288 application, suggesting that defective *I*_h_ contributes, at least partly, to the neuronal hyperexcitability observed in the PRMT7 KO CA1 neurons.

To verify the role of PRTM7 in the control of HCN channels within the CA1 pyramidal neurons, we assessed the HCN channel activities in the PRMT7-knockdown primary hippocampal neurons. Specifically, we tested whether PRMT7 knockdown affects the hyperpolarization-induced depolarizing “sag”, a measure of *I*_h_ activity, in a current clamp configuration^39^. In Supplementary Fig. 2, injection of the hyperpolarizing current step (100 pA) elicited substantial sag depolarizations in the control but not in the PRMT7 knockdown neurons (Supplementary Fig. 2a). The mean normalized sag amplitude was significantly larger in the control neurons than in the PRMT7 knockdown neurons (Supplementary Fig. 2b). These data further indicate that PRMT7 depletion reduces *I*_h_ activity.

### PRMT7 regulates *I*_h_ in the CA1 pyramidal cells via the control of HCN1 protein expression

**Fig. 4:**
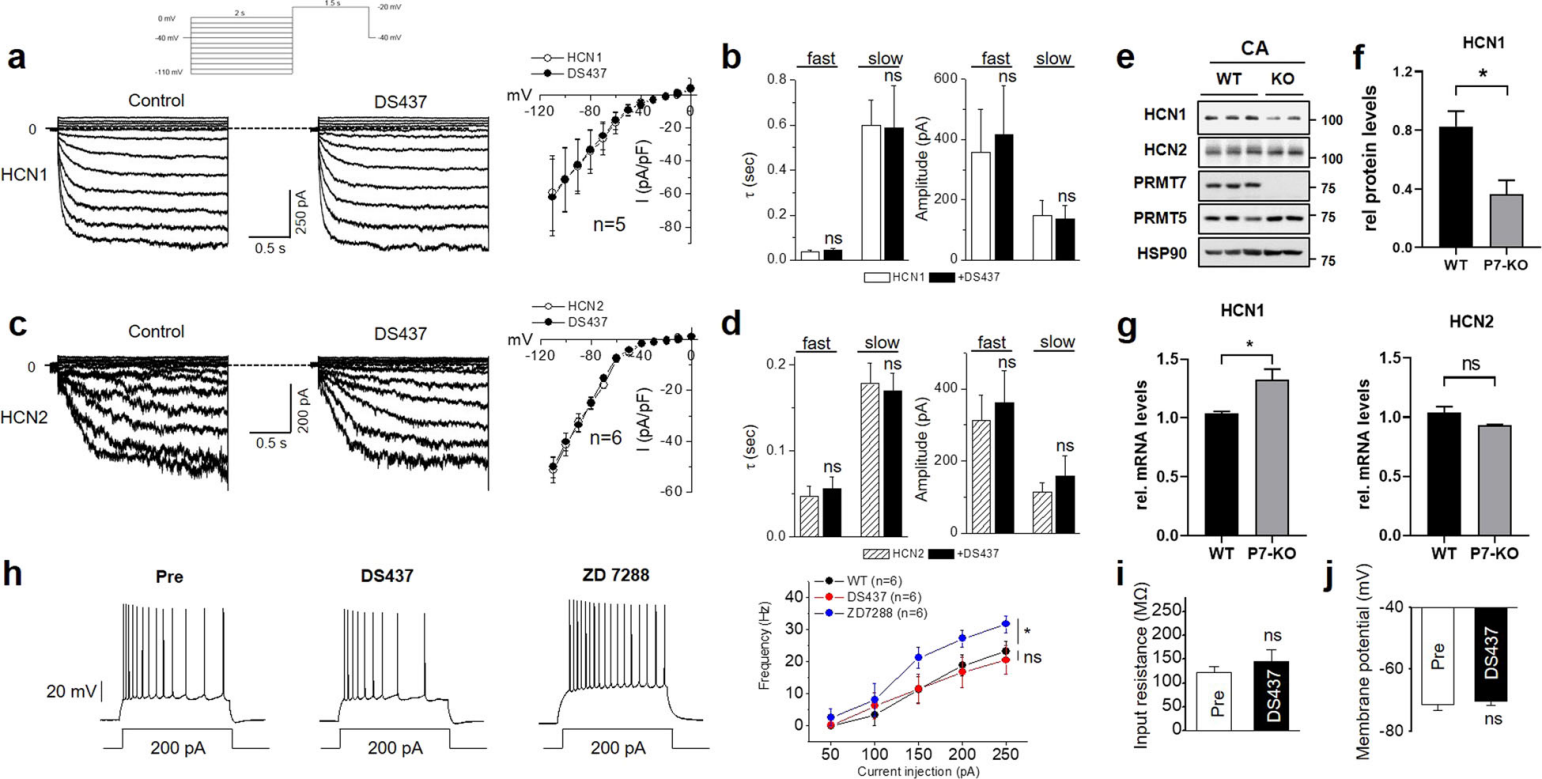
PRMT7 regulates *I*_h_ in the CA1 neurons by controlling HCN1 protein expression. **a**, **c** Representative current traces recorded in the range from 0 to −110 mV (10-mV steps; holding potential −40 mV) in the HEK-293T cells expressing HCN1 (**a**) or HCN2 (**c**) channels before and after 10 min of 100 μM DS437, as indicated. Right, graphs showing the mean steady-state current density–voltage relations. Inset, experimental protocol. **b**, **d** Summary graphs of time constants (*τ*) and amplitudes of fast and slow components of *I*_h_-current activation at a test potential of −110 mV for HCN1 (**b**) and HCN2 (**d**). **e** Immunoblot analysis for HCN1, HCN2, PRMT5, and PRMT7 in the WT and PRMT7 KO CA region. HSP90 was used as a loading control. **f** Quantification of the HCN1 protein levels in **e**. Data represent the mean ± S.D., Mann–Whitney test for comparison, *p < 0.05. **g** qRT-PCR analysis of the HCN1 and HCN2 mRNA levels in the CA region of brains from the 8-week-old WT and PRMT7 KO animals. *n* = 3. Data are presented as the mean ± S.E.M. Mann–Whitney test for comparison with the control cells. ns, not significantly different, *p < 0.05; Student’s *t* test. **h**–**j** Comparisons of excitability in the CA1 pyramidal cells before and after 10 min of application of 100 μM DS437. **h** illustrates representative traces before and after the application of DS437 and the subsequent application of ZD7288. Right panel, the mean number of action potentials (AP no.) plotted against the eliciting currents. At all amplitudes, the mean ± S.E.M. input resistance and resting membrane potential are shown in **i** and **j**, respectively. *p < 0.05; ns not significantly different; Student’s *t* test.

We then investigated the detailed mechanism underlying the reduced *I*_h_ in the PRMT7-deficient CA1 neurons. Channel activity is defined as *NP*_o_ (a product of the channel number (*N*) and the open probability (*P*_o_)). We first investigated whether PRMT7 regulates *I*_h_ via modulation of the open probabilities. We reconstituted *I*_h_ by expressing HCN1 or HCN2 in HEK-293T cells, which do not have important endogenous leak currents, and measured the channel function before and after a specific blocker of PRMT5 and PRMT7, DS437 (Fig. 4a–d). The *I*_h_ currents were readily activated by 2-s hyperpolarizing voltage steps, exhibited a reversal potential of −32 mV, and were inhibited by extracellular Cs^+^ and ZD7288 (data not shown). As described previously^40^, the two HCN subunits produced *I*_h_ currents that substantially differed in the activation properties; HCN2 currents activated more slowly than HCN1 currents (Fig. 4a, c). The brief bath application of DS437 had little effect on the HCN1 or HCN2 currents in the HEK-293T cells; current density, the shape of the current–voltage (*I*−*V*) relationships, and the kinetics of *I*_h_-current activation for the HCN1 and HCN2 currents were not altered (Fig. 4a–d).

As the activation kinetics and the open probabilities remained unchanged by PRMT7 inhibition, one likely explanation for the reduction of *I*_h_ in the PRMT7 KO CA1 pyramidal cells is a decrease in the HCN protein expression. This hypothesis was tested by measuring the levels of the HCN1 and HCN2 proteins in the hippocampal CA tissue samples from the adult WT and PRMT7 KO mice (Fig. 4e). The protein expression of HCN1 in the PRMT7 KO hippocampal CA was reduced compared with that in the WT hippocampal CA. A densitometric analysis showed that, compared to those in the WT mice, the HCN1 protein levels in the hippocampal CA of the PRMT7 KO mice were decreased by 50% (Fig. 4f) (one-sample *t* test, *p* = 0.002). However, the protein level of HCN2 in the PRMT7 KO hippocampal CA did not differ from that in the WT CA (Fig. 4e). Thus the magnitude of the reduction in the HCN1 protein level in the PRMT7 KO hippocampal CA region closely paralleled the reduction in *I*_h_. We further examined whether this reduction in the HCN1 protein level is caused by the decreased transcript level of HCN1 in the PRMT7 KO CA1 cells. The HCN1 and HCN2 transcription levels were not decreased in the PRMT7 KO CA (Fig. 4g). These data suggest that PRMT7 depletion causes a reduction in the HCN1 protein levels. As SHANK3, a well-known ASD-causing gene, is a major determinant of the HCN protein level in hippocampal neurons^39^, we examined whether PRMT7 might regulate HCN channels via alteration of SHANK3 gene expression. This hypothesis was tested by measuring the SHANK3 protein levels in the hippocampal CA tissues from the adult WT or PRMT7 KO mice. The protein expression of SHANK3 in the PRMT7 KO hippocampal CA region was reduced compared with that in the WT hippocampal CA region (Supplementary Fig. 3a). We further examined whether this reduction in the SHANK3 protein level is caused by the decreased transcript level of SHANK3 in the PRMT7 KO hippocampal CA region. The SHANK3 transcription levels were significantly decreased in the PRMT7 KO CA region (Supplementary Fig. 3b). In addition, we measured SHANK3 in the control or the PRMT7 shRNA-expressing HEK-293T cells. PRMT7 knockdown led to a significant reduction in both the protein and transcript levels of SHANK3 compared to those of the control cells (Supplementary Fig. 3c, d). These data suggest that PRMT7 KO induces a decrease in SHANK3 gene expression, which may contribute to HCN channel impairment.

If the reduction in the HCN1 protein levels is the underlying mechanism for the decreased *I*_h_ in PRMT7 KO neurons, a brief application of DS437 to the WT CA1 pyramidal neurons should not trigger any changes in the AP firing rates and the passive membrane properties produced by PRMT7 KO. In contrast to genetic inhibition, short-term pharmacological inhibition of PRMT7 with 100 μM DS437 (10 min) did not affect the excitability, input resistance, or membrane potential of the CA1 pyramidal cells (Fig. 4h–j). We thus concluded that PRMT7 deficiency reduces *I*_h_ activities via a reduction in HCN1 expression in the CA1 pyramidal cells, leading to increased neuronal excitability and impairment of social behavior.

## Discussion

Recent studies of several neuropsychiatric disorders, including ASDs and their animal models, have led to the hypothesis that social and communication deficits are associated with neuronal hyperexcitability in the hippocampal CA1 pyramidal neurons^15,41,42^. Despite this association between CA1 and social deficits, the underlying disturbances in the CA1 region remain largely unexplored. Our data showed that the deletion of the PRMT7 gene in mice causes an impairment in social behaviors. Concomitantly, neuronal excitability of the CA1 pyramidal cells was significantly increased in the PRMT7 KO mice. We revealed that PRMT7 KO impairs *I*_h_-channel function in the CA1 pyramidal cells as a primary impairment, which in turn produces major changes in intrinsic neuronal excitability.

Electrophysiological analysis showed that PRMT7 deficiency led to an increase in the firing frequency and the input resistance, leading to hyperpolarization of the RMP in the CA1 pyramidal cells. We found that *I*_h_-current inhibition with ZD7288 in the WT CA1 pyramidal cells also significantly increased the firing frequency and the input resistance, and these effects were very similar to those of PRMT7 KO. In contrast to the results in the WT CA1 pyramidal cells, the addition of ZD7288 to the PRMT7 KO CA1 neurons triggered no further effects. In contrast to the effects observed in the PRMT7 KO neurons, a brief exposure to the PRMT7 inhibitor DS437 had little effect on the current amplitudes and activation kinetics of *I*_h_ mediated by HCN1 or HCN2 after overexpression in HEK-293T cells as well as neuronal excitability of the CA1 pyramidal cells. These results indicated that PRMT7 inhibition does not affect the open probabilities of the HCN channels. Molecular analysis demonstrated the colocalization of HCN1 and HCN2 with PRMT7 in the CA1 region in the WT mice, and PRMT7 deficiency in CA1 causes reduced HCN1 protein levels without alteration in the HCN2 protein levels or reduction in the HCN1 mRNA levels. Given that channel activity is defined as *NP*_o_ (a product of the channel number and the open probability), these data suggest that PRMT7 deficiency leads to decreased *I*_h_ activities through reduced HCN1 protein levels. The reduction in the HCN1 protein level was well correlated with the decreased expression of SHANK3, a scaffolding protein for the HCN channel. In addition, SHANK3 modulates the HCN protein levels by direct interaction, and SHANK3 mutations predispose individuals to autism by inducing dysfunction of HCN channels. Since PRMT7 knockdown led to a significant reduction in both the protein and transcript levels of SHANK3 in the HEK-293T cells, we hypothesize that PRMT7 deficiency induces an alteration of SHANK3 expression that contributes to the decreased protein level of HCN1. However, our results do not exclude the possibility that the activity or abundance of additional families of voltage or ligand-activated ion channels, or a combination thereof, may be altered by PRMT7.

The fact that PRMT7 inhibition can increase neuronal excitability has three important implications. First, from a pathoetiological perspective, our findings suggest new mechanistic hypotheses for neuropsychiatric disorders associated with alterations in neuronal excitability. Disorders characterized by alterations in neuronal excitability might have underlying dysregulation in the expression of HCN channels and associated proteins mediated by altered methylation. This mechanism is certainly possible for ASDs^16^ but may be valid in other disorders, such as anxiety^43,44^, depression^14^, and epilepsy^45^. Second, interventions or manipulations that rely on broad spectrum or even isoform-specific modulation of PRMT7 function will affect neuronal excitability in CA1. For example, if alterations in intrinsic excitability are critical to the pathogenesis of a specific disorder, modulation of PRMT7 could be a potential therapeutic strategy. If not, approaches that target arginine methylation of specific ion channels would be preferred to limit unintentional consequences on neuronal excitability.

Together with a previous study^24^, our data indicate that PRMT7 targets distinct classes of ion channels to control neuronal function in a cell-type-specific manner. PRMT7 preferentially regulates NALCN channels in DG granule cells but not the HCN channel current in CA1 pyramidal cells. Both the NALCN and HCN channels contribute to the RMP of neurons and control neuronal function. NALCN depolarizes neurons and increases intrinsic excitability. However, HCN currents not only depolarize the RMP but also exert a shunting effect on neurons. Thus the net effect of the depolarizing and shunting properties of *I*_h_ on excitability is combinatorial and depends on many factors^46^. Consistent with previous studies showing that *I*_h_ currents have anti-excitatory effects in hippocampal pyramidal neurons^16,47^, our data showed that blockade of this channel current causes an increase in neuronal excitability in the CA1 pyramidal cells. The neuron-type-specific targets of PRMT7 might depend on its subcellular localization in specific types of neurons. PRMTs display different subcellular localizations in different cell types, indicating cell- and tissue-specific mechanisms for regulating PRMT function^48^. Similarly, distinct distribution patterns of ion channels exist in the somata, dendrites, and axons of specific cell types and brain regions, as well as during development^49–53^. For elucidation of the exact mechanism underlying these factors, however, further studies will be required.

Taken together, our data suggest that an *I*_h_-current reduction is a key feature associated with the hyperexcitability of the CA1 pyramidal cells in the PRMT7 KO mice. A role for HCN channelopathy as a mechanism for impaired social behavior in the PRMT7 KO mice is plausible given the association of HCN channels with human neuropsychiatric disorders, including ASDs^54–60^. However, we cannot rule out that the hyperexcitability of DG granule cells might also contribute to social deficits in these mice. Nevertheless, *I*_h_-channel function can conceivably be influenced by protein arginine methylation, providing a platform for the design of novel therapeutic strategies for ASDs and other neuronal hyperexcitability disorders.

## Supplementary information

Supplementary information
