# Supplementary material for: PRMT7 deficiency causes dysregulation of the HCN channels in the CA1 pyramidal cells and impairment of social behaviors
Source: Exp Mol Med. 2020 Apr 8;52(4):604–14. doi: 10.1038/s12276-020-0417-x (PMC7210990; doi:10.1038/s12276-020-0417-x)
Supplement: Supplementary file 1 — Supplementary information [file 12276_2020_417_MOESM1_ESM.docx]

**Supplementary Figure legends**

**Supplementary Fig. 1. PRMT7 KO mice do not exhibit spontaneous seizures. a** representative 30 s electroencephalogram (EEG) traces in WT (top) and PRMT7^-/-^ mice (bottom). **b, c** Mean bar graphs of seizure spike frequency (**b**) and number of seizures per day (**c**) in WT (n = 10) and PRMT7 KO mice (n = 10).

**Supplementary Fig. 2. Effects of PRMT7 knockdown on I_h_ activity in primary hippocampal neurons. a** Quantitative RT-PCR analysis for mRNA expression level of *Prmt7* in control (shCTL) and PRMT7-knockdown (shPRMT7) primary hippocampal neurons. **b** Sample recordings showing a voltage sag generated by I_h_ in response to the hyperpolarizing current step at -100 pA. PRMT7-knockdown primary hippocampal neurons displayed a decrease in the amplitude of the sag response. **c**. Summary of the voltage sag between control (n = 3) and PRMT7-knockdown neurons (n = 5). **p < 0.01; Student’s t-test.

**Supplementary Fig. 3. Effects of PRMT7 deficiency on SHANK3 gene expression. a** Immunoblot analysis for SHANK3, PRMT5, and PRMT7 in CA of 8-week-old mice brain. **b** Reduced mRNA expression of SHANK3 in 8-week-old mice brain of PRMT7^-/-^ (KO) CA compared to PRMT7^+/+^ (WT) control mice. **c** Immunoblot analysis for SHANK3 and PRMT7 in control or PRMT7 knockdown HEK-293T cells. **d** Quantitative RT-PCR analysis for mRNA expression level of *Shank3* and *Prmt7* in control or PRMT7 knockdown HEK-293T cells.

**Fig. S1**

**
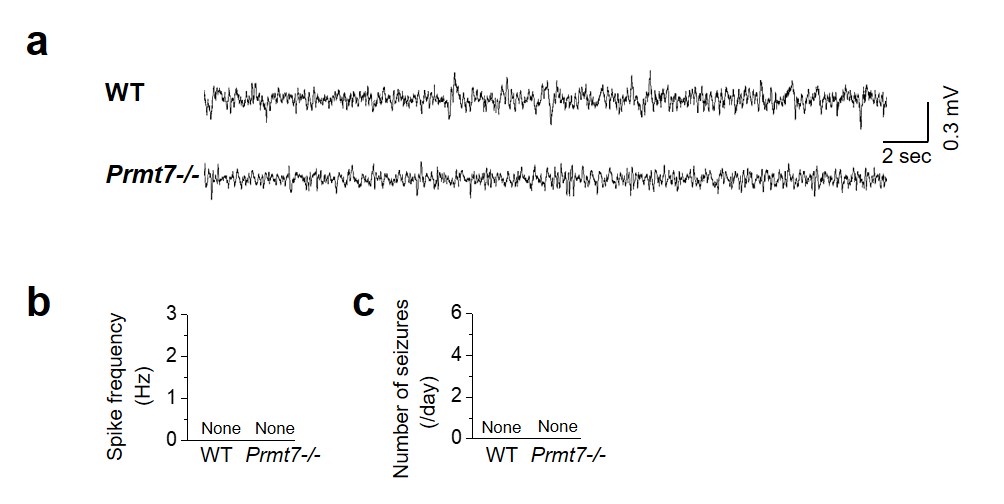
**

**Fig. S2**

**
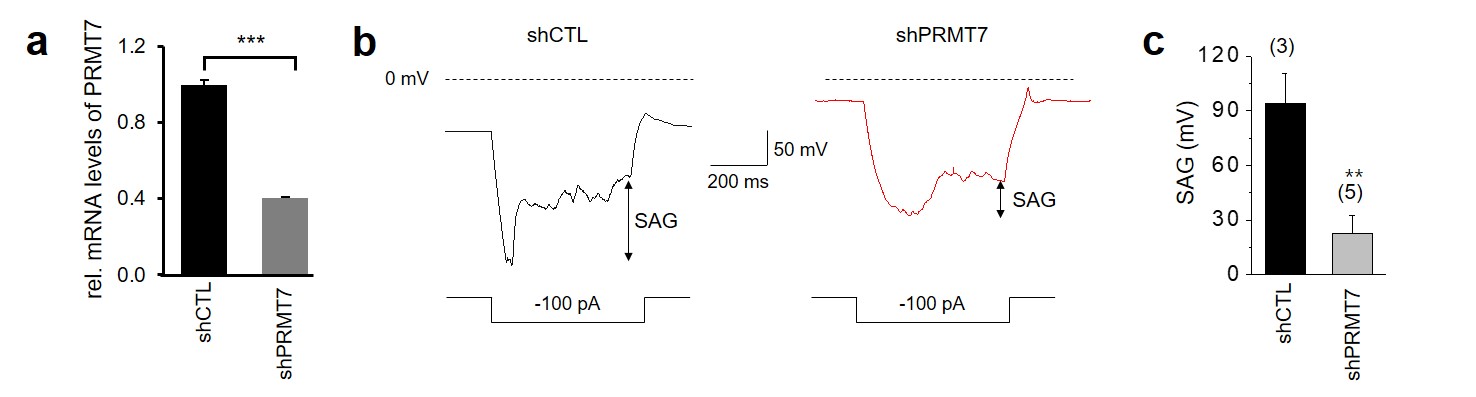
**

**Fig. S3**

**
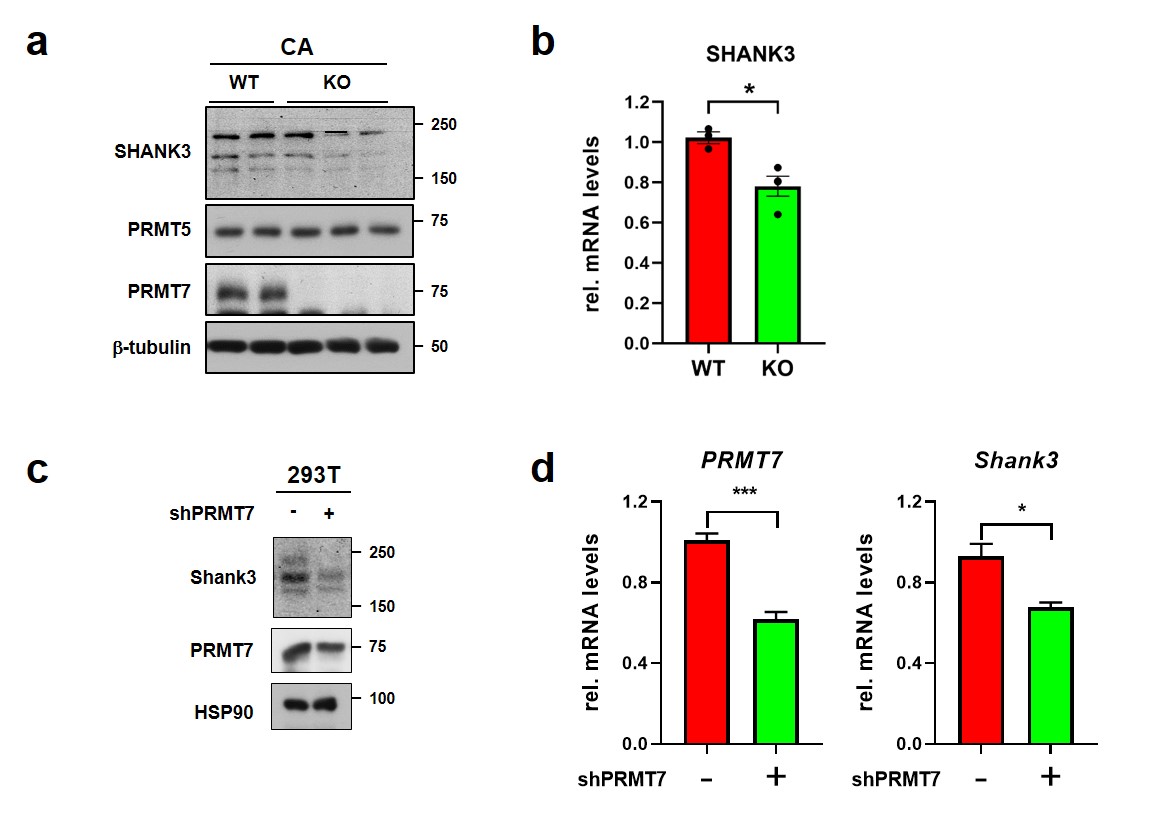
**
